# Co-Stimulation-Impaired Bone Marrow-Derived Dendritic Cells Prevent Dextran Sodium Sulfate-Induced Colitis in Mice

**DOI:** 10.3389/fimmu.2018.00894

**Published:** 2018-05-03

**Authors:** Carl Engman, Yesica Garciafigueroa, Brett Eugene Phillips, Massimo Trucco, Nick Giannoukakis

**Affiliations:** ^1^Institute of Cellular Therapeutics, Allegheny Health Network, Pittsburgh, PA, United States; ^2^Department of Biological Sciences, Carnegie Mellon University, Pittsburgh, PA, United States

**Keywords:** dendritic cells, immune hyporesponsiveness, autoimmunity, tolerogenic dendritic cells, regulatory immune cells, regulatory B-cells, regulatory T-cells, retinoic acid

## Abstract

Dendritic cells (DC) are important in the onset and severity of inflammatory bowel disease (IBD). Tolerogenic DC induce T-cells to become therapeutic Foxp3+ regulatory T-cells (Tregs). We therefore asked if experimental IBD could be prevented by administration of bone marrow-derived DC generated under conventional GM-CSF/IL-4 conditions but in the presence of a mixture of antisense DNA oligonucleotides targeting the primary transcripts of CD40, CD80, and CD86. These cell products (which we call AS-ODN BM-DC) have demonstrated tolerogenic activity in preventing type 1 diabetes and preserving beta cell mass in new-onset type 1 diabetes in the NOD mouse strain, in earlier studies. In addition to measuring efficacy in prevention of experimental IBD, we also sought to identify possible mechanism(s) of action. Weight, behavior, stool frequency, and character were observed daily for 7–10 days in experimental colitis in mice exposed to dextran sodium sulfate (DSS) following injection of the AS-ODN BM-DC. After euthanasia, the colons were processed for histology while spleen and mesenteric lymph nodes (MLNs) were made into single cells to measure Foxp3+ Treg as well as IL-10+ regulatory B-cell (Breg) population frequency by flow cytometry. AS-ODN BM-DC prevented DSS-induced colitis development. Recipients of these cells exhibited significant increases in Foxp3+ Treg and IL-10+ Breg in MLN and spleen. Histological examination of colon sections of colitis-free mice remained largely architecturally physiologic and mostly free of leukocyte infiltration when compared with DSS-treated animals. Although DSS colitis is mainly an innate immunity-driven condition, our study adds to the growing body of evidence showing that Foxp3+ Treg and IL-10 Bregs can suppress a mainly innate-driven inflammation. The already-established safety of human DC generated from monocytic progenitors in the presence of the mixture of antisense DNA targeting the primary transcripts of CD40, CD80, and CD86 in humans offers the potential to adapt them for clinical IBD therapy.

## Introduction

While the major target for dendritic cell (DC) therapy, relying on their powerful immunostimulatory ability, has been malignancy (1), the long-sought objective of using the other aspect of DC which is their capability to induce immune hyporesponsiveness, clinically took its first step forward in the last decade in a phase I safety trial humans (2). In addition to this first-in-concept trial for type 1 diabetes, accumulating encouraging preclinical data using different embodiments of tolerogenic DC to treat various other autoimmune conditions have made possible a number of other clinical trials. These include trials in the space of rheumatoid arthritis, multiple sclerosis, and intestinal bowel disease (3). Even though the different DC populations differ in the methods used to generate them *ex vivo*, what they appear to share in common is a mechanism that results in the increase in T-regulatory cells, a feature that is inherent in naturally occurring DC that are shaped *in vivo* into a tolerogenic state.

Endogenous DC are mainly found in the immature state and orchestrate tolerance largely by maintaining and promoting the frequency and activity of mainly CD4+ CD25+ regulatory T-cells (Tregs) (4). Immature, co-stimulation impaired DC are known to actively induce the differentiation and proliferation of Foxp3+ Tregs (4–11) [reviewed in Ref. (12, 13)]. This mechanism underlies peripheral tolerance to autoantigens and hyporesponsiveness to alloantigens in transplantation studies (7–11, 14, 15). Co-stimulation-impaired DC and DC engineered to produce cytokines promoting Foxp3+ Tregs successfully prevent, attenuate, and reverse autoimmunity and facilitate allograft survival (7–11, 14, 15).

We showed for the first time that DC generated from monocytic progenitors in the presence of the mixture of antisense DNA targeting the primary transcripts of CD40, CD80, and CD86 were safe in humans (2). In addition, data from this first-in-human trial demonstrated that some recipients of these DC began to exhibit C-peptide positivity during and slightly beyond the cell treatment cycle. This is noteworthy given that these patients were C-peptide negative during screening and baseline testing. Whether this could anticipate potential benefits is currently unknown and will have to be established in phase II trials.

One of the notable characteristics of the DC generated from monocytic progenitors in the presence of the mixture of antisense DNA targeting the primary transcripts of CD40, CD80, and CD86 used in the phase I type 1 diabetes safety trial is their ability to produce retinoic acid (RA) (16, 17). RA and other retinoids have been shown to regulate autoimmunity in rheumatoid arthritis, experimental encephalomyelitis, and type 1 diabetes (18–20). RA, acting *via* the RA receptor, affects the transcription of Foxp3, IL-17, and RORγt, thereby participating in the local homeostasis of Tregs through the balance of Tregs:TH17 cells (21, 22). RA, in fact, has been shown to attenuate experimental colitis by increasing the numbers of Tregs and inhibiting the generation of TH17 cells (22, 23). RA-producing DC are, in fact, naturally found in the mucosa (24, 25), and their role is suggested to be one of maintenance of a stable immunoregulatory state preventing the exacerbation of gut inflammation (24, 25). There is evidence that such RA-producing DC also express CD103 and, at least in the mucosa and more recently in the pancreas, CD103+ DC exert a tolerogenic effect (26–29) even though they can be immunostimulatory under specific conditions (30–33). Tolerogenic DC that express CD103 act *via* their ability to induce Foxp3 expression in T-cells (28, 34–42), especially in the presence of TGF-β in an RA-dependent manner (22, 43–46). Under homeostatic conditions, gut CD103+ DC constitutively migrate to the mesenteric lymph node (MLN) (47). Gut CD103+ DC preferentially support antigen-induced spontaneous differentiation of Foxp3+ Tregs from naive precursors. Furthermore, CD103+ DC isolated from the MLN of ovalbumin-fed mice activate and drive naive DO11.10 CD4+ T cells to express Foxp3 (48). Intestinal CD103+ DC were shown to efficiently differentiate *in situ* into tolerogenic DC (43–45, 48, 49). Thus, adoptive immunotherapy for inflammatory bowel disease (IBD) could become clinically relevant since DC that prevent and reverse T1DM exhibit features similar to gut tolerogenic CD103+ DC; they are stably immature, co-stimulation-impaired, and express the RA-metabolizing enzyme ALDH1A2 which together convert immunosuppressive progenitors of Foxp3+ Tregs into highly suppressive Foxp3+ Tregs.

Various approaches to generate tolerogenic DC for use in mouse models of IBD have been demonstrated. Curcumin treatment of *in vitro*-generated bone marrow-derived DC resulted in the expression of ALDH1 as well as IL-10 and these DC, acting *via* induction of Tregs and Tr1 cells, inhibited colitis *in vivo* (50). Pedersen et al. used IL-10-conditioned bone marrow-derived DC exposed to an enterobacterial extract to suppress colitis severity and weight loos in SCID mice adoptively transferred with CD4+ CD25− colitogenic T-cells (51). Vasoactive intestinal peptide-conditioned bone marrow DC showed efficacy in the TNBS model of murine colitis (52). This study was the first to show that anatomic area selection for DC administration was relevant in facilitating the accumulation of the DC into the MLNs, where the most important antigen presentation and activation of Th1/Th17 cells takes place (53). A popular approach to generating tolerogenic DC has been the combination dexamethasone/vitamin D3 conditioning of bone marrow DC (54–57), and these DC were shown to suppress colitis in the CD4+ CD25− colitogenic T-cell transfer SCID model (58). Although these antigen-agnostic approaches were effective, some studies suggest that provision of IBD-relevant antigen improves therapeutic outcomes [e.g., by provision of carbonic anhydrase I; (59)].

Although these studies were concurrent with our research in the area of type 1 diabetes, as well as a phase I clinical trial using dexamethasone-generated autologous DC in refractory Crohn’s disease having been initiated (http://clinicaltrials.gov identifier NCT02622763), given that bone marrow-derived DC generated in the presence of a mixture of antisense DNA oligonucleotides targeting the CD40, CD80, and CD86 primary transcripts (which we term *AS-ODN BM-DC*) shown to mobilize Tregs and regulatory B-cells (Bregs) in the NOD mouse strain, cells that are critical in maintaining tolerance also in the intestinal tissues, we considered that AS-ODN BM-DC could also be useful to treat IBD, and more importantly in a severe model of murine colitis. The additional rationale to consider our these DC is also underlied by the data showing their production of RA (16) which was a contributing factor to the differentiation of B-cells into IL-10+ Bregs and the proliferation of existing IL-10+ Bregs (16). Given the accumulated evidence that Bregs are also potent regulators of colitis (60–64), that the deficiency of Bregs in mice results in exacerbated arthritis with increased frequency of TH17 cells and decreased Foxp3+ Tregs (65), we have now tested the efficacy of AS-ODN BM-DC to treat IBD using the dextran sodium sulfate (DSS) colitis mouse model and to determine the degree of Treg and/or Breg involvement.

## Materials and Methods

### Animals

All mice were maintained in a specific pathogen-free environment, and experiments were conducted in line with specific protocols approved by the Allegheny Health Network IACUC.

### Human Blood

We purchased human complete blood from a commercial source (Grifols) from which we generated DC (see below). The blood products were obtained from a normal adult individual with no reported acute or chronic health conditions or disease.

### Generation of Murine DC

Two types of DC were generated for the purposes of this research endeavor: (i) DC from bone marrow progenitors (which we term BM-DC) and (ii) DC from bone marrow progenitors that were cultured in the presence of a mixture of antisense DNA oligonucleotides targeting the CD40, CD80, and CD86 primary transcripts (which we term *AS-ODN BM-DC*). Both DC populations were generated from bone marrow progenitors from 7- to 8-week-old C57BL/6 mice (Bar Harbor, ME, USA) in 6-day cultures with GM-CSF and IL-4 using previously published protocols (BM-DC) (66, 67). The DC generated in the continuous presence of a mixture of phosphorothioate DNA oligonucleotides targeting the primary transcripts of CD40, CD80, and CD86 (AS-ODN BM-DC) are immunosuppressive. The cells generated at the end of the 6-day culture in the presence of only GM-CSF and IL-4 (BM-DC; no antisense oligonucleotides) are mostly DC; however, there are some undifferentiated monocytic precursors. To generate the AS-ODN BM-DC, the same antisense oligonucleotide sequences and backbone chemistry used in the study by Machen et al. were used in this study (67). BM-DC served as control cell populations in this study. The phenotype and characteristics of the AS-ODN BM-DC have been published elsewhere (66–69). Prior to adoptive transfer of the AS-ODN BM-DC into mice, and for each such experiment, we verified that the general phenotype and functionality of these cells conformed to that which we have previously shown [(67); i.e., low cell surface expression of CD40, CD80, CD86, and the ability to suppress the proliferation of allogeneic leukocytes *in vitro*]. For this, we compare the mean fluorescence intensity of CD40, CD80, and CD86 in day 6 AS-ODN BM-DC to BM-DC using flow cytometry (see below). Table S1 in Supplementary Material also provides other characteristics of BM-DC and AS-ODN BM-DC. To determine the functional phenotype of BM-DC and AS-ODN BM-DC, we added splenocytes from freshly isolated spleen of allogeneic mice (Balb/c) to BM-DC or AS-ODN BM-DC-containing IFNγ ELISPOT assay plates (ELISPOT-PLUS, MabTech) for 72 h as recommended by the manufacturer. Results of these two verifications, representative of routine outcomes, are shown in Figures S1A,B in Supplementary Material.

### DSS Colitis/Treatment of Mice With BM-DC or AS-ODN BM-DC

Following a standard DSS induction protocol (70, 71), mice were randomly placed into three groups (*n* = 4 mice per group; two independent study cohorts totaling *n* = 8 mice per treatment group): DSS, DSS+ BM-DC recipients, and DSS+ AS-ODN BM-DC recipients. Three days prior to exposure to DSS, mice were injected with 2 × 10^6^ BM-DC or AS-ODN BM-DC intraperitoneally (i.p.) in a minimal volume of sterile endotoxin-free PBS or the PBS vehicle only as control. All mice were then switched to drinking water containing 3.5% DSS to which they had *ad libitum* access for 5 days. On day 3 of exposure to DSS, a second injection of 2 × 10^6^ moDC, iDC, or PBS vehicle i.p. was administered. Mice were euthanized 7–10 days after the initiation of DSS exposure.

### Measurements/Assessment of Colitis

Mice were weighed on the day before DSS exposure and then every day thereafter until euthanasia. Colitis was assessed by weight loss, stool consistency, fecal blood, and anal prolapse. Upon euthanasia, colons were harvested, flushed, and fixed for histopathological and immunofluorescence assessment. Concurrently, the MLNs and spleen were collected, made into single cells in preparation for flow cytometric measurements.

### Flow Cytometry

FACSCalibur/FACSAria with DIVA support (BD Biosciences) or Influx workstations with species-specific antibodies, non-overlapping fluorophores, and appropriate isotype controls were used for flow-sorting and FACS analyses. Cells were antibody stained either after pre-enrichment for specific populations over magnetic columns (Miltenyi Biotec) or stained as freshly isolated single cells from MLNs or spleen *in vitro*.

To measure Tregs, we used the detection system that includes the FJK-16s Foxp3-specific antibody, CD4-FITC clone RM4-5, and CD25-APC clone PC61.5 (eBioscience). For B-cell population characterization and FACS analysis, the following antibodies were used (all from BD Biosciences): B220 (clone RA3-6B2), CD19 (clone 1D3), CD5 (clone 53-7.3), and CD1d (clone 1B1). IL-10-producing cells were identified following positive selection along IL-10 surface adsorption using a commercial magnetic isolation method (Miltenyi Biotec product #130-090-435, Auburn, CA, USA). Characterization of these cells as Bregs was then confirmed by FACS with the B-cell antibodies listed above.

To measure the frequency of DC producing RA, with or without the expression of CD103, we first stained single splenocytes or MLN cells with the ALDEFLUOR reagent (StemCell Technologies, BC, Canada) (72, 73) with parallel control cell cultures treated with *N*,*N*-diethyl-amino-benzaldehyde (DEAB), an inhibitor of all ALDH isozymes and therefore endogenous background non-specific fluorescence. Subsequently, we stained with a CD103-specific antibody (clone 2E7, Biolegend, CA, USA) and measured the frequency of CD103+ ALDEFLUOR+ cells by flow cytometry. True ALDEFLUOR fluorescence was taken as the measurement in the ALDEFLUOR reagent-treated cells minus the measurement in the DEAB-treated cells.

BM-DC and AS-ODN BM-DC accumulation inside the MLNs following i.p. injection was measured post-administration of the cells pulsed *in vitro* with fluorescent nanoparticles (Fluospheres; Thermo Fisher). Cells were injected within 5 h of confirmed nanoparticle uptake. 3–72 h later, the MLNs were harvested and single cells were stained with fluorescence-tagged CD45 (clone 30-F11, BD Biosciences) and CD11c (clone Rea754, Miltenyi Biotec) antibodies. The percentage of fluorescent nanoparticle+ cells inside a CD45+ CD11c+ gate was considered to represent the number of exogenously administered BM-DC or AS-ODN BM-DC that accumulated into the tissue.

Prior to adoptive transfer into mice, CD40, CD80, and CD86 surface levels on BM-DC and AS-ODN BM-DC were measured using the following antibody clones directly conjugated with non-overlapping excitation/emission fluors: CD40 (clone 3/23), CD80 (clone 16-10A1), and CD86 (clone GL1). These antibodies were purchased from BD Biosciences (San Jose, CA, USA) and titered before use.

### Histology/Immunocytochemistry

The colons of mice were cut into proximal, middle, and distal segments. After fixation in 4% paraformaldehyde (Sigma-Aldrich, MO, USA) for 3–4 h, tissues were transferred to 30% sucrose (Sigma-Aldrich, MO, USA) overnight, and then embedded in Tissue-Tek OCT (Fisher Chemicals, NJ, USA). 10-µm frozen sections were cut. For H&E staining, frozen sections were dried at room temperature, and staining was then conducted with a commercially available kit (Frozen Section Staining Kit; Thermo Fisher Scientific, NJ, USA). For H&E-based inflammation assessment, each colon segment was scored individually, and these scores were summed to reach a total score for the entire colon. Histological scores were assigned as follows: 0, normal; 1, ulcer or cell infiltration limited to the mucosa; 2, ulcer or limited cell infiltration in the submucosa; 3, focal ulcer involving all layers of the colon; 4, multiple lesions involving all layers of the colon, or necrotizing ulcer larger than 3 mm in length. Thus, the total possible histologic score is 12. Scoring was performed by a pathologist blinded to the treatment of the mouse.

### Detection of Human IL-37 in DC Culture *In Vitro*

Two populations of DC were generated from freshly obtained PBMC of a healthy volunteer as described previously (16). One population of DC was generated in the presence of GM-CSF/IL-4 and served as a control cell population. The other was generated in the presence of GM-CSF/IL-4 (which we term conventional PBMC DC; CP-DC) and a mixture of antisense DNA oligonucleotides targeting the primary transcripts of CD40, CD80, and CD86, which we term tolerogenic human DC (TH-DC). These two DC populations were used in a phase I clinical trial in established type 1 diabetic patients and shown to increase the frequency of human Bregs *in vivo* and *in vitro* (2, 16) *via* RA production (16). 1 × 10^5^ CP-DC or TH-DC were cultured for 18 h in the presence or absence of 2 μg/mL LPS. The culture supernatants were collected and IL-37 was detected by a human-specific ELISA (R&D Systems, catalog # DY1975). The concentration of the cytokine in cell-free serum-containing medium was taken to represent control.

### Statistical Analyses

Two-tailed *t*-tests were used to determine the statistical relevance of the differences in the means of *in vitro* outcomes where replicates were considered (e.g., replicate cell culture wells in multi-well plates). When comparing the differences between two groups of mice, one-tailed ANOVA with Dunnett’s *post hoc* test was conducted or repeated-measures Kruskal–Wallis test, depending on the experimental objective. Differences in the colitis score in the colons of different groups of mice was determined by one-way MANOVA.

A *p* value of <0.05 was considered to indicate statistical relevance to the differences in the outcomes in all statistical tests listed above.

## Results

### BM-DC and AS-ODN BM-DC Prevent DSS-Induced Colitis

**Figure 1:**
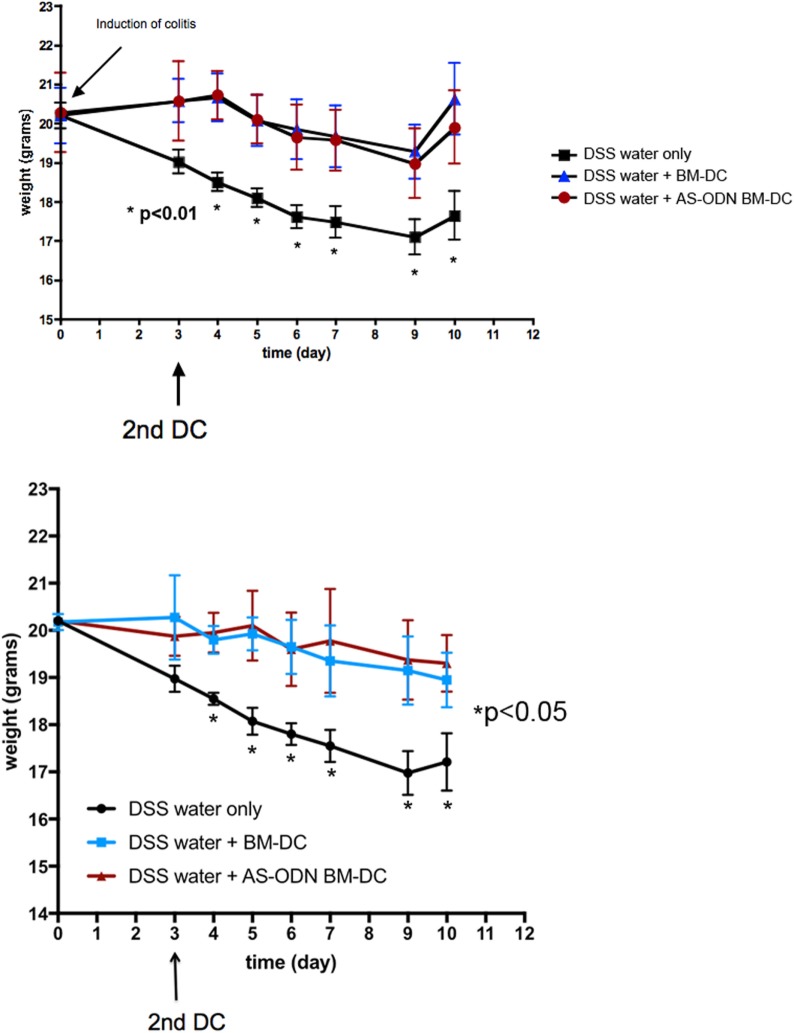
BM-dendritic cell (DC)/AS-ODN BM-DC pretreatment followed by a second injection 3 days after dextran sodium sulfate (DSS) colitis induction attenuates weight loss in mice. The graph shows the median weight (solid symbols) of DSS-exposed mice that were injected with BM-DC, AS-ODN BM-DC, or PBS vehicle 3 days before DSS exposure followed by a second DC (or PBS vehicle) injection 3 days following DSS exposure. Each graph shows the outcome in three groups of four mice. The bars represent the SD of *n* = 4 mice in each treatment group. Two mouse cohorts independently treated are shown. At each time point, represented in the graphs by an asterisk, the difference in weights between the BM-DC/AS-ODN BM-DC treatment arms, and the control mouse arm was statistically significant (determined by one-tailed ANOVA with Dunnett’s *post hoc* test; *p* < 0.01 in study cohort 1, top graph panel, and *p* < 0.05 in study cohort 2, bottom graph panel). BM-DC indicates treatment of mice with GM-CSF and IL-4-generated cells from bone marrow progenitors and AS-ODN BM-DC indicates treatment with BM-DC generated in the presence of GM-CSF/IL-4 with the antisense DNA oligonculeotides.

In Figure 1, we show the median weights and the range (error bars) of the mice in each of the three DSS treatment groups (no DC, BM-DC, and AS-ODN BM-DC). These observations were consistent among the two treatment cohorts which represented two independently conducted experiments. Those mice that were not treated exhibited significant weight loss and typical symptoms associated with DSS colitis (evidence of blood in feces as well as anal prolapse). By contrast, the AS-ODN BM-DC and BM-DC treatments were effective in significantly preventing weight loss. There was no statistically distinguishable difference in the outcomes in mice treated with AS-ODN BM-DC or BM-DC. We did not observe blood in stools in the DC-treated mice.

### Increased Frequency of Foxp3+ Tregs in Colitis-Free DC Recipients

**Figure 2:**
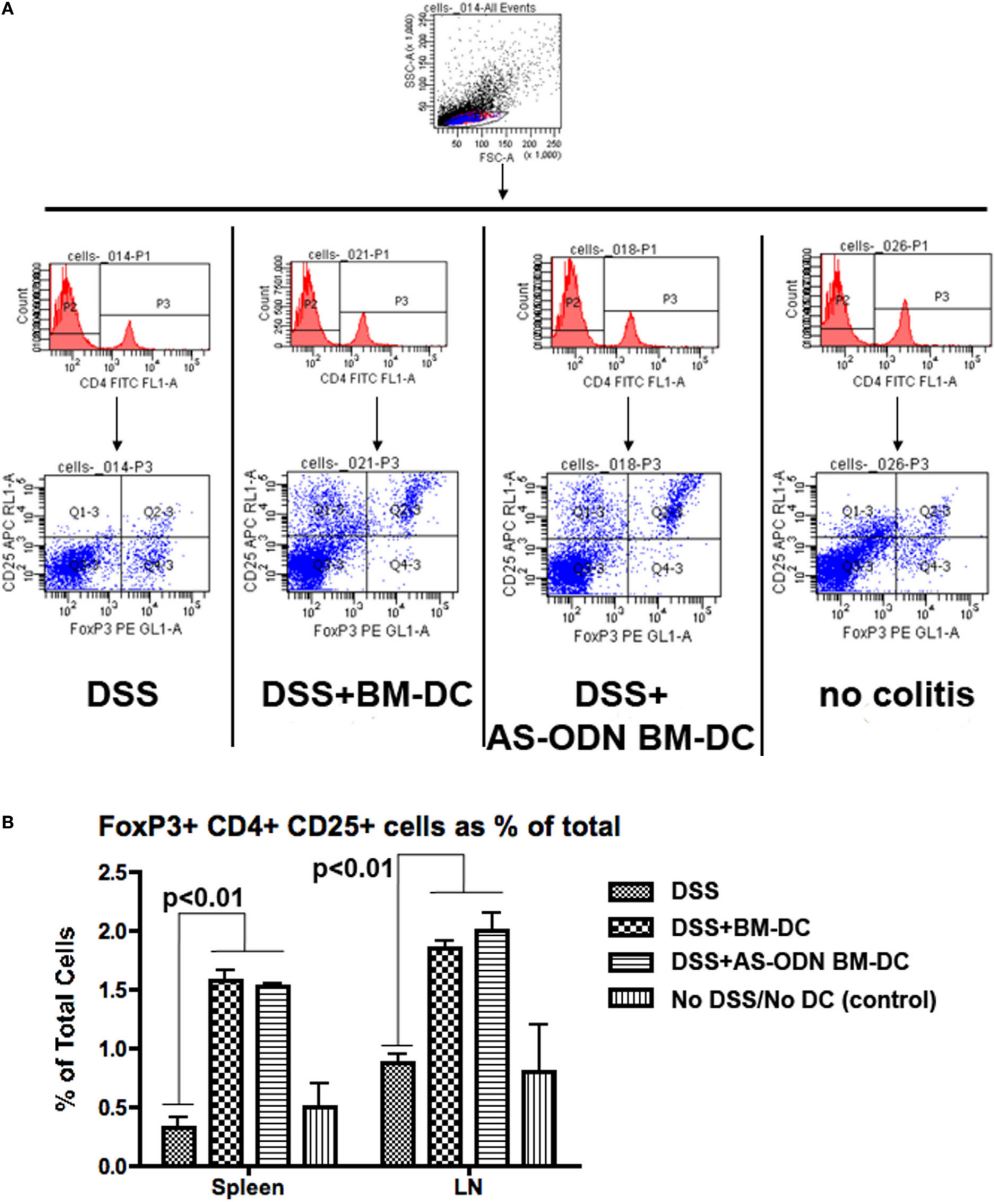
Increased frequency of Foxp3 regulatory T-cells (Tregs) in the spleen and the mesenteric lymph node (MLN) of BM-dendritic cell (DC) and AS-ODN BM-DC-treated mice exposed to dextran sodium sulfate (DSS). **(A)** The figure outlines the gating strategy for the flow cytometry analysis to measure CD4+ CD25+ Foxp3+ Tregs. The data are representative of the measurements in the spleens of four mice of all treatment groups (DSS: DSS exposure alone; DSS+ BM-DC; BM-DC pretreatment prior to DSS and then a second injection 3 days later; DSS+ AS-ODN BM-DC: AS-ODN BM-DC pretreatment prior to DSS and then a second injection 3 days later; and control: no DSS exposure, injection of PBS vehicle). Quadrant 2–3 of the bottom panels represents the channels inside which CD25+ Foxp3+ cells were measured after gating for CD4 positivity (middle panels). **(B)** The graph summarizes the frequency of Foxp3+ Tregs in the spleens and MLN of DSS-exposed mice alone (No DC); DSS-exposed and BM-DC-injected mice; DSS-exposed and AS-ODN BM-DC-injected mice; and untreated control mice (No DSS/No DC). The bars represent the means of Foxp3+ Tregs as a % of total cells (splenocytes or lymph node cells) and the error bars the SEM. For both spleen and MLN, the difference in the means between the BM-DC/AS-ODN BM-DC and control mice (DSS alone or untreated) were statistically significant (*p* < 0.01, one-way ANOVA).

Given the evidence that tolerogenic DC promote the differentiation of T-cells into Foxp3+ Tregs while preventing conversion of gut T-cells into effector TH17-type cells (48, 74, 75), we hypothesized that the beneficial outcomes of the AS-ODN BM-DC treatment in the DSS-exposed mice could be associated with increased Foxp3+ Treg in the MLN and possibly other lymphoid organs into which the exogenously injected DC could potentially accumulate. In Figure 2, we demonstrate that Foxp3+ Tregs are increased in frequency as a % of total cells in the MLN. The analysis shown in Figure 2 was conducted on cells obtained from tissue collected 5 days following DC administration. The increase in cell number was evident as early as 3 days following DC administration (data not shown). Similar results were obtained when measuring the frequency of Tregs in spleen from identically treated mice (Figure 2B). There were no apparent differences in frequency of Tregs in the analyzed tissues between mice treated with AS-ODN BM-DC or BM-DC.

### Increased Frequency of B10 Bregs in Colitis-Free DC Recipients

**Figure 3:**
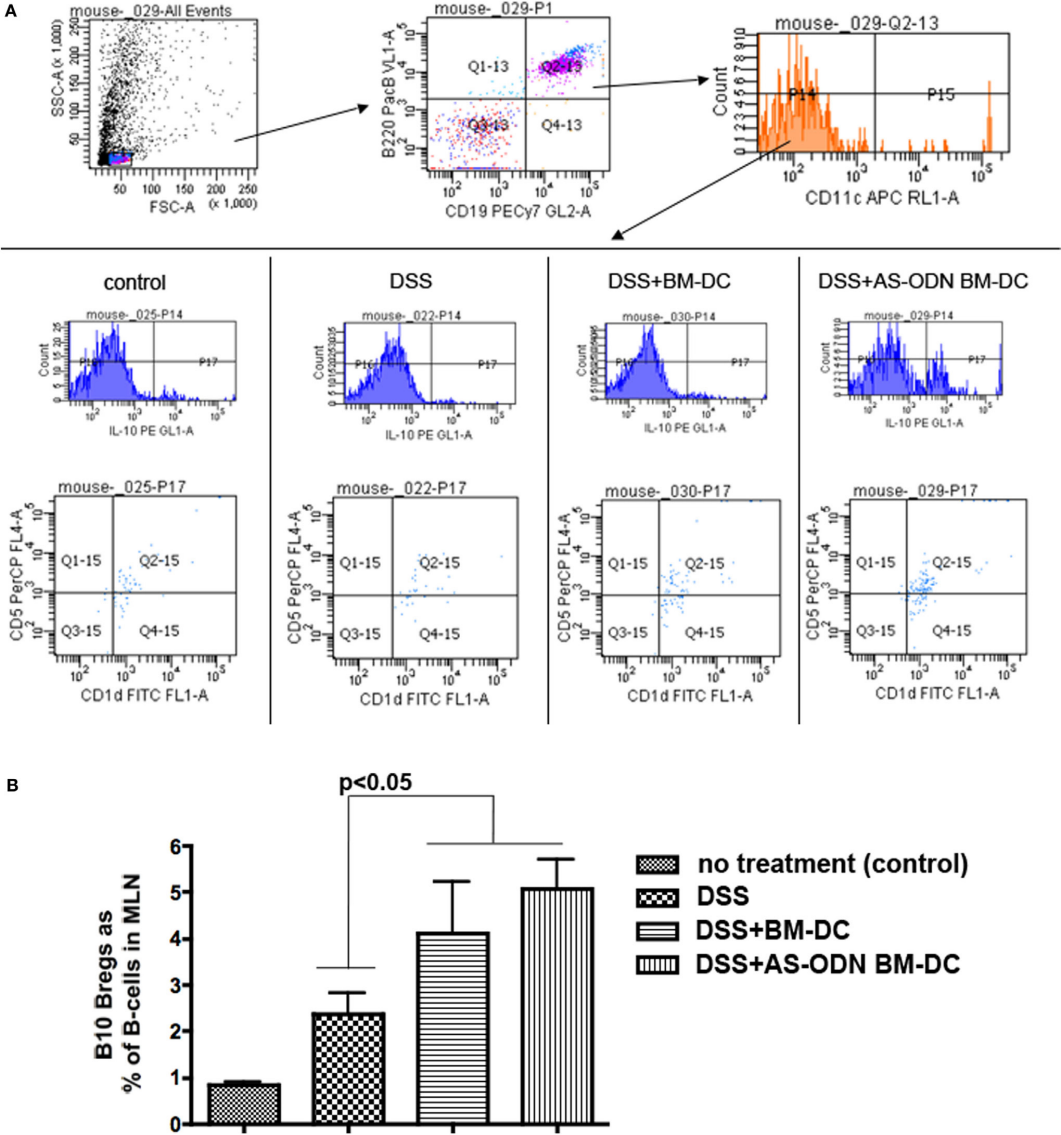
Increased frequency of B10 regulatory B-cells (Bregs) in the mesenteric lymph node (MLN) of BM-dendritic cell (DC) and AS-ODN BM-DC-treated mice exposed to dextran sodium sulfate (DSS). **(A)** The panels show the gating strategy for flow cytometric analysis to measure B220+ CD19+ CD11c− IL-10+ CD1d+ CD5+ B-cells (B10 Bregs). The data are representative of the frequency of these cells in the MLN in four mice of each of the indicated treatment groups (control: no DSS exposure, injection of PBS vehicle; DSS: DSS exposure alone; DSS+ BM-DC; BM-DC pretreatment prior to DSS and then a second injection 3 days later; DSS+ AS-ODN BM-DC: AS-ODN BM-DC pretreatment prior to DSS and then a second injection 3 days later). Quadrant 2–15 of the bottom panels indicates the channels inside which CD1d+ CD5+ cells were measured after sequential gating for: (i) B220+ CD19+ positivity and (ii) CD11c negativity; and then IL-10 positivity (top and middle panels). **(B)** The graph summarizes the frequency of B10 Bregs in the MLN of DSS-exposed mice alone (colitis); DSS-exposed and BM-DC-injected mice; DSS-exposed and AS-ODN BM-DC-injected mice; and untreated control mice (no colitis). The bars represent the means of CD1d+ CD5+ IL-10+ B220+ CD19+ CD11c− cells as a % of B220+ CD19+ B-cells and the error bars the SEM. The difference in the means between the BM-DC/AS-ODN BM-DC and control mice (DSS alone or untreated) were statistically significant (*p* < 0.05, one-way ANOVA).

Accumulating data indicate that B-cells can act in a suppressive manner and a number of these B-cells, although with some differences in phenotype (76, 77), can transfer protection and improve experimental arthritis, lupus, and colitis in mice (78–80). We have presented evidence that immature DC, including our AS-ODN BM-DC, directly increase the prevalence of the “B10” Breg population (79, 80) *in vitro* and *in vivo* (16, 17). We measured the frequency of B10 Bregs in the MLN and the spleen of mice pre-treated with BM-DC and AS-ODN BM-DC prior to DSS colitis induction. In Figure 3, we show that B10 Bregs increased in frequency as a % of total B-cells (% of CD19+ B220+ cells) in MLN but not in spleen (data not shown). In fact, DC treatment had no effect on the frequency of B10 Bregs in spleen of any treatment group, including DSS induction on its own (data not shown). The analysis shown in Figure 3 was conducted on cells obtained from tissue collected 5 days following DC administration. The increase in cell number was evident as early as 3 days following DC administration (data not shown). Even though there are no apparent differences in the frequency of Bregs in the tissues analyzed between AS-ODN BM-DC and BM-DC recipients, on a per-cell basis, the density of IL-10 in the AS-ODN BM-DC recipients was significantly greater than that in the BM-DC recipients (Figure S2 in Supplementary Material).

### BM-DC and AS-ODN BM-DC Accumulate Inside the MLNs After i.p. Injection

To confirm that BM-DC and AS-ODN BM-DC accumulate inside the MLNs of DSS-treated mice, we pulsed the DC with fluorescent nanoparticles *in vitro* (Fluospheres). Within 5 h of pulsing, a time when a maximal number of nanoparticles was phagocytosed by the DC, the cells were resuspended in sterile PBS and injected i.p. In Figure S3 in Supplementary Material, we show that BM-DC and AS-ODN BM-DC accumulated inside the MLNs as early as 3 h following administration (shown in figure). Accumulation was maximal by 3 days (data not shown). There were no statistically distinguishable differences in MLN-accumulated cells between BM-DC and AS-ODN BM-DC recipients.

### Increased Frequency of CD103+ ALDEFLUOR+ DC in Colitis-Free DC Recipients

**Figure 4:**
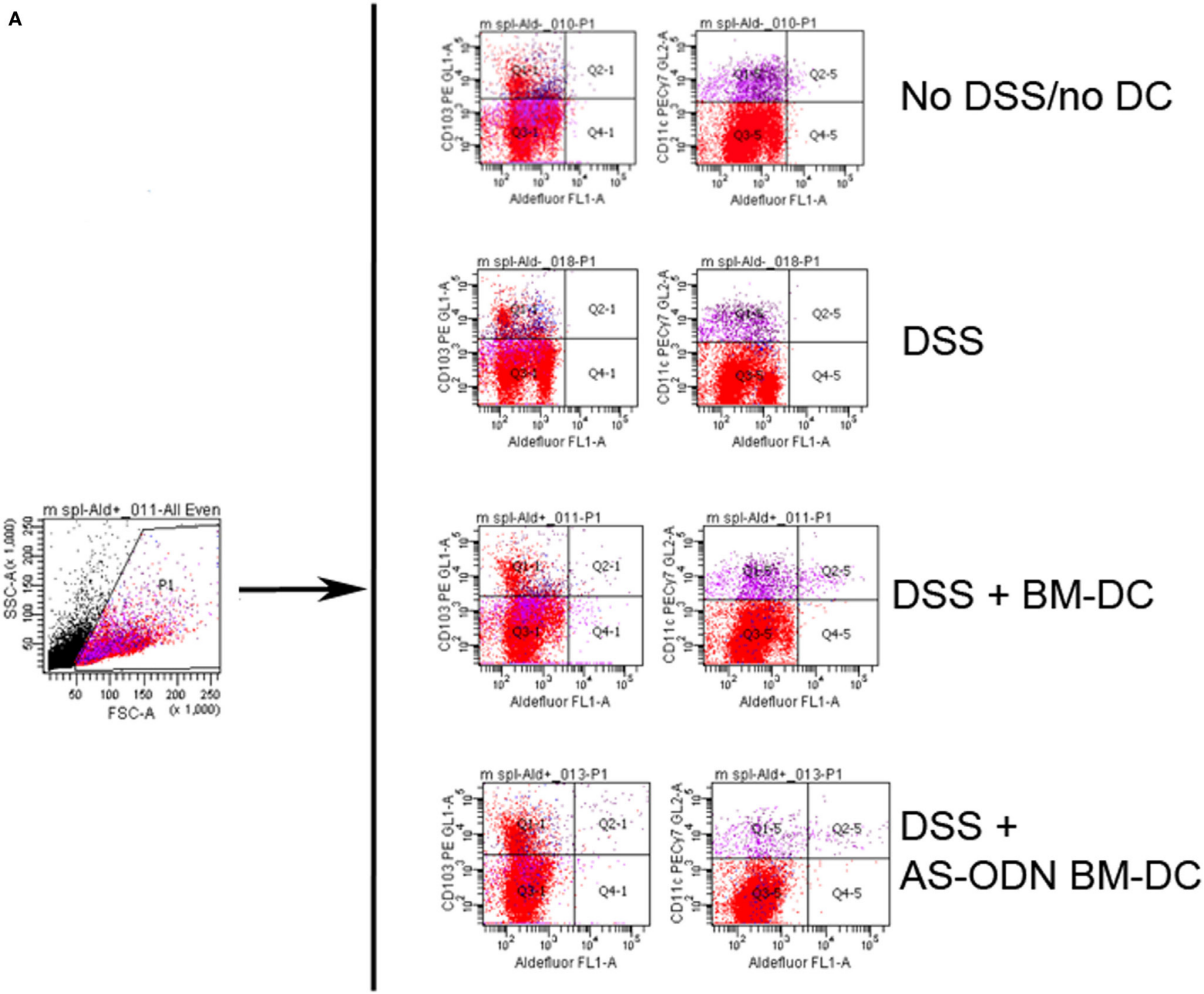

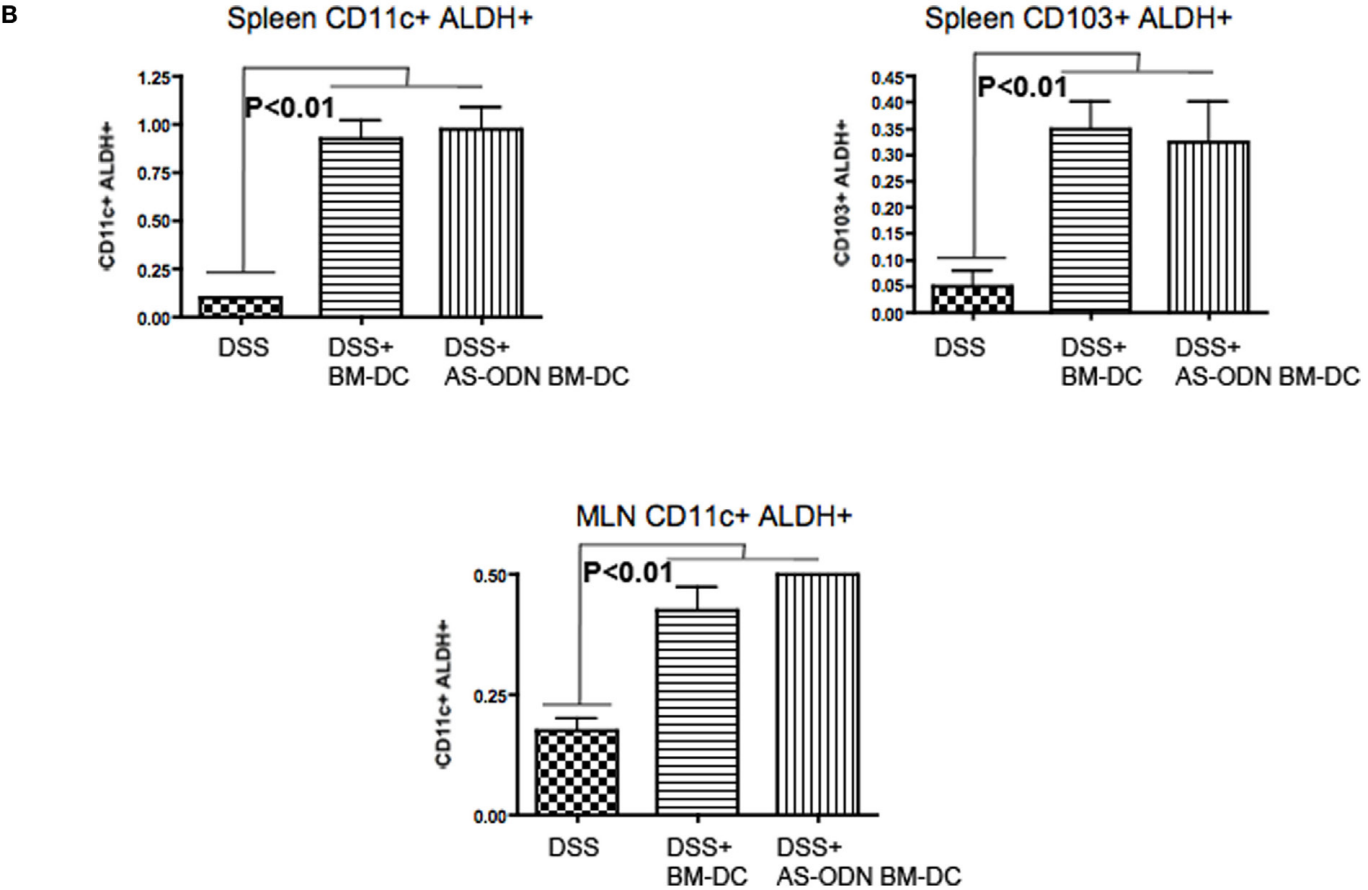
Increased frequency of retinoic acid (RA)-producing dendritic cell (DC) in the spleen and mesenteric lymph node (MLN) of BM-DC and AS-ODN BM-DC-treated mice exposed to dextran sodium sulfate (DSS). **(A)** The figure outlines the gating strategy for the flow cytometric analysis to measure CD103+ or CD11c+ cells that produce RA (i.e., that are reactive with the ALDEFLUOR reagent; ALDEFLUOR+ cells). The data are representative of the measurements in the spleen and MLN in four mice of each of the indicated treatment groups (DSS: DSS exposure alone; DSS+ BM-DC; BM-DC pretreatment prior to DSS and then a second injection 3 days later; DSS+ AS-ODN BM-DC: AS-ODN BM-DC pretreatment prior to DSS and then a second injection 3 days later). Quadrant 2–1 of the middle panels represents the channels inside which CD103+ ALDEFLUOR+ cells were measured and Quadrant 2–5 represents the channels inside which CD11c+ ALDEFLUOR+ cells were measured. Fluorescence in DEAB-treated cells was ascertained inside the same channels and used as a negative control (data not shown). **(B)** The graph summarizes the frequency of CD11c+ ALDEFLUOR+ cells in the spleen and MLN as well as the CD103+ ALDEFLUOR+ cells in the spleens of DSS-exposed mice alone (colitis); DSS-exposed and BM-DC-injected mice; and DSS-exposed and AS-ODN BM-DC-injected mice. CD103+ cells were detectable only in spleens of even untreated mice and not in the MLN. ALDH+ indicates ALDEFLUOR-reactive cells after subtraction of the background fluorescence using the DEAB inhibitor. The bars represent the means of the double-positive cells as a % of total splenic and MLN cells and the error bars the SEM. The difference in the means between the BM-DC/AS-ODN BM-DC and control mice (DSS-exposed) were statistically significant (*p* < 0.01, one-way ANOVA).

Although BM-DC and AS-ODN BM-DC express ALDH and produce RA *in vitro* (16, 17), we hypothesized that exogenous administration of these DC could change the endogenous DC phenotype in the spleen and the MLN of treated mice. We therefore measured the frequency of total DC expressing ALDH (CD11c+ ALDEFLUOR+) as well as the frequency of CD103+ ALDEFLUOR+ cells as a function of total splenocytes or MLN single cells in DSS colitis mice treated with BM-DC or AS-ODN BM-DC. In Figure 4, we show that CD11c+ ALDEFLUOR+ cell frequency was significantly increased in mainly the AS-ODN BM-DC-recipients. The differences in CD103+ ALDEFLUOR+ cells between BM-DC or AS-ODN BM-DC and no DC recipients were statistically significant in the splenic population (bottom graph, Figure 4B). Although we observed similar differences in ALDEFLUOR+ CD11c+ cells in the MLN, we were unable to verify the presence of CD103+ cells that co-stained consistently with ALDEFLUOR in the MLN of these mice (data not shown).

### Colitis-Free DC Recipients Exhibit Inflammation-Attenuated Colon Architecture

**Figure 5:**
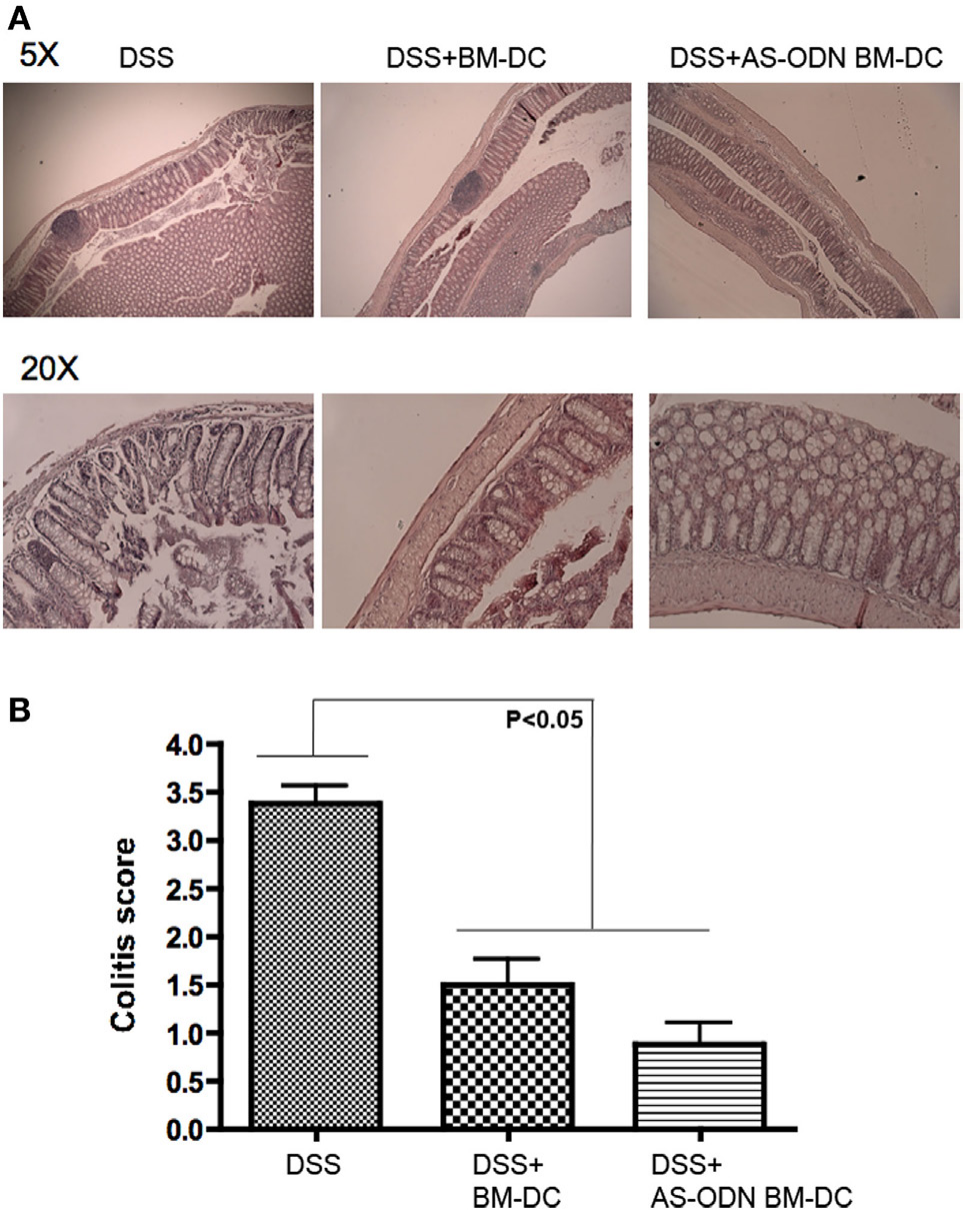
AS-ODN BM-DC treatment preferentially preserves colon architecture with significant protection from inflammation in dextran sodium sulfate (DSS)-exposed mice. **(A)** H&E staining of colons resected from DSS-exposed mice treated with BM-dendritic cell (DC) or AS-ODN BM-DC. Representative sections are shown at two magnifications (×5 and ×20). Untreated, DSS-exposed mice exhibit inflammatory as well as significant tissue architecture disruption. Even though BM-DC treatment does not prevent inflammatory foci formation, the architecture of the tissue remains mostly intact. AS-ODN BM-DC treatment preserves colon architecture with evidence of significant protection from inflammation. **(B)** Colitis inflammation in resected colons of DSS-treated mice administered BM-DC or AS-ODN BM-DC was scored in a blinded manner. The bars in the graph represent the mean score of all colon sections (representing four mice per treatment arm) assessed. The error bars show the SEM. The differences in scores between the BM-DC/AS-ODN BM-DC and control (DSS colitis) mouse colons were statistically significant (*p* < 0.05, one-way MANOVA).

H&E staining of representative sections of tissue from control, BM-DC, and AS-ODN BM-DC-treated mice suggested that DC significantly attenuated inflammation (Figure 5A). In Figure 5B, we summarize the scoring of inflammation in all treated mice.

## Discussion

Many studies confirm the tolerogenic capacity of immature DC (81–83). Clinical applications of these DC have long been sought for transplantation tolerance and as a method to treat autoimmunity; however, the stability of the immature state *in vivo*, once the cells have been administered, has acted as a conceptual barrier to clinical translation. Our successful phase I clinical trial in established T1DM human volunteers with co-stimulation impaired, tolerogenic DC (2), together with the outcomes of more recent clinical safety trials using other variations of tolerogenic DC (84–86) should compel a reassessment of this barrier. In preclinical and ongoing studies in the NOD mouse model of T1DM, as well as a number of transgenic strains, we have discovered that DC (human and mouse) generated in the presence of antisense DNA oligonucleotides targeting the CD40, CD80, and CD86 primary transcripts increase the frequency of suppressive immune cells including Foxp3+ Tregs (66, 67, 69) and novel Bregs (16, 17). Based on the evidence demonstrating that RA-generated Tregs are therapeutic for IBD and that tolerogenic DC producing RA upregulate the number of Foxp3+ Tregs, we predicted that AS-ODN BM-DC (2, 67) could be beneficial in IBD as well. It is worth noting that BM-DC are inherently immature and immunosuppressive on their own. The rationale behind our approach to generate these DC in the presence of the antisense oligonucleotides targeting CD40, CD80, and CD86 was to ensure that these major co-stimulation proteins are stably knocked down *in vivo*. Unconditioned BM-DC, exogenously administered into an inflammatory environment such as that in T1DM, can rapidly differentiate into potent immunostimulatory cells characterized by high-level surface expression of CD40, CD80, and CD86 (87, 88).

In previous studies, we demonstrated that AS-ODN BM-DC stimulate the proliferation of existing Bregs concomitantly with the differentiation of B-cells into Bregs *in vivo* in NOD mice (17) and we also showed that the human counterpart to the AS-ODN BM-DC population generated from peripheral blood monocyte progenitors (TH-DC) also achieved the same outcome *in vitro*, in human co-cultures (16). Herein, we implicate B10 Bregs as responsive to BM-DC and AS-ODN BM-DC administration *in vivo* in the DSS colitis model of IBD. Although B-cells have been traditionally viewed as effector-type immune cells, mainly producing antibody and serving as accessory antigen-presenting cells, accumulating evidence supports their immunosuppressive ability. IL-10 production appears to be a defining feature of immunosuppressive B-cells. Two major populations of B-cells uniquely adapted to act as specific regulatory, immunosuppressive cells have been identified and characterized (89, 90). Even though IL-10 expression is the main feature of these Bregs, its production is not a *conditio sine qua non* for immunosuppression as we and others have reported elsewhere (91, 92) (and unpublished observations). Bregs, especially the B10 population suppress inflammation in experimental autoimmune encephalomyelitis, collagen-induced arthritis, and colitis (93–95). In a spontaneous model of murine colitis, the prevalence of B10 Bregs increases at the peak of inflammation and suppresses the disease by attenuating IL-1 and STAT3-mediated processes of immune reactivity (93). In another model of colitis, in TCR-alpha-deficient transgenic mice, B-cell deficiency exacerbates disease and only CD40 ligand-activated B-cells can adoptively transfer protection and suppress the colitis inflammation (96). Although it is not yet clear where Bregs act to suppress the inflammation, evidence suggests that B-cells isolated from MLN are stable suppressors of colitis, even though splenic marginal zone B-cell exhibit a plasticity of suppressive ability when adoptively co-transferred with Gαi2-deficient CD3+ T-cells into Rag2-deficient mice (97). Interestingly, in murine models of colitis as well as in lupus, very few marginal zone splenic B-cells are found within the inflammation area further supporting a lymph node-source of suppressive B-cells. Our data are compatible with such a possibility where stably suppressive Bregs within the MLN are mobilized following their interaction with tolerogenic DC. Alternatively, endogenous, intralymphatic DC differentiate into tolerogenic DC upon encounter with the exogenously administered DC, in an RA-dependent manner. That Foxp3+ Tregs and B10 Bregs are increased in frequency coordinately inside the MLN following BM-DC and AS-ODN BM-DC administration (which produce RA) leads us to propose a model whereby DC are central in converting T-cells and B-cells into suppressive cells which then migrate into the inflamed colon structures to prevent or attenuate inflammation. This model is in the process of being tested in our laboratory.

Although the underlying mechanisms of B10 Breg and Foxp3+ Treg increase in the spleen and MLN are currently unclear in these experiments in the DSS colitis model, previous studies have outlined two non-mutually exclusive pathways concerning DC-stimulated increases in Treg numbers. DC can directly promote the proliferation of naturally occurring Tregs inside the lymph nodes (98–100). However, a second mechanism appears to be more common and this involves the conversion of resting naive T-cells that either do not express, or express low levels of Foxp3, into suppressive Tregs (101). These adaptive, or induced, Tregs exhibit some plasticity in suppressive ability and depending on the presence or absence of cytokines like IL-10 or TGF-β, can revert to non-suppressive cells (101). RA and TGF-β coordinately provide a third mechanism, especially in IBD, effectively blocking the conversion of naive T-cells in the periphery into TH17-type cells and instead directing them into potently suppressive Foxp3+ Tregs (74, 75, 102). Accumulating data in our lab suggest that BM-DC and AS-ODN BM-DC (AS-ODN BM-DC > BM-DC) administration results in increased Foxp3 immunoreactivity through the entire colon tissue (unpublished observations). Together with the increased splenic and MLN complement of Foxp3+ Tregs, a significant tolerogenic state is established *in vivo* and this, along with the increase in B10 Bregs, could be a powerful suppressant of the most acute and damaging experimental model of colitis; DSS. Although it is not currently clear in the data from the study herein, how AS-ODN BM-DC compel an increase in the frequency of Bregs, our mechanistic studies in NOD mice could provide some insight (16, 17). There, AS-ODN BM-DC stimulate the proliferation of existing Bregs together with the differentiation of B-cells into Bregs. We propose that similar mechanisms underlie the AS-ODN BM-DC effect in the DSS colitis model, although these will have to be formally demonstrated in ongoing studies. If, and how the DC treatment affects less acute and less disruptive IBD models (e.g., adoptive transfer of CD4+ CD25− T-cells into SCID mice) remains to be determined. That AS-ODN BM-DC suppressed the severity of DSS colitis, which is mainly a non-T-cell-driven inflammatory syndrome, raises the intriguing possibility that tolerogenic DC can suppress the ability of innate immune cells to cause autologous tissue pathology and to even impede their ability to stimulate adaptive immune responses. This is not unprecedented (103, 104). Also, in an already-discovered and described mechanism, tolerogenic DC-stimulated Tregs can directly affect the function of innate immune cells including in IBD (105–118). Another, more recently described mechanism could involve IL-37. We propose that AS-ODN BM-DC (and their human counterpart generated from peripheral blood monocytic progenitors) could suppress DSS colitis through either or all of the above mechanisms. For example, they can directly interfere with innate immune cell activity (e.g., ability to produce chemokines), they can stimulate the proliferation and differentiation of Tregs and Bregs that can then go on to impede innate immune cell activity, and they could even trigger the production of IL-37 in a proinflammatory environment. IL-37 will then act directly on innate immune cells, possibly *via* the IL-18 signaling pathway, to dampen their activity. This would affect the severity of both innate-driven inflammation and the impedance of triggering of the adaptive arm. This also is not without precedent. Luo et al. have shown that DC expressing IL-37 are tolerogenic (119) and subsequent to that discovery, Dinarello and colleagues demonstrated the suppressive effects of IL-37 on innate inflammation (120). Indeed, in preliminary experiments, we have discovered that human DC generated from PBMC in the presence of GM-CSF/IL-4 (CP-DC) as well as CP-DC generated with the addition of the mixture of the antisense DNA oligonucelotides targeting the primary transcripts of CD40, CD80, and CD86 (TH-DC; refer to Section “Materials and Methods”) produce IL-37 (Figure S4 in Supplementary Material). Interestingly, when stimulated with LPS, CP-DC produced less IL-37 in culture, however, when TH-DC were stimulated with LPS the amount of IL-37 produced was slightly greater (albeit not statistically significant; Figure S4 in Supplementary Material). It is worth noting that IL-37 production *in vitro* and very likely *in vivo* may exhibit interindividual variation and initial observations suggest that this is so (BP, CE, MT, NG; manuscript in preparation). Whether such variation could be associated with autoimmunity is unknown. Since stimulation of T-cell differentiation into Tregs and/or proliferation of existing Tregs by AS-ODN BM-DC cannot account for the rapid effects we observe in the DSS colitis model, we hypothesize that the second mechanism of innate immune suppression potentially underlies our observations in the DSS colitis model. This possibility is currently under investigation.

Onji and colleagues have reported that carbonic anhydrase I-pulsed bone marrow-derived DC generated *in vitro* with GM-CSF/IL-10/TGF-β inhibited colitis progression *via* rebalancing the Foxp3+/TH17 T-cell ratio inside the MLN (59). Pedersen and colleagues have also shown that tolerogenic DC pulsed with fecal extract suppressed colitis development (51). Early data suggest that, in addition to Foxp3 Tregs, cecal bacterial extract-pulsed DC protect from experimental colitis by generating Tr1 Tregs (52, 121). Our findings agree with those of these investigators, with the benefit that our approach does not rely on antigen-pulsing or preconditioning of the DC with immunosuppressive cytokines. More importantly, our AS-ODN BM-DC have been successfully translated clinically (using leukapheresis-sourced monocytic progenitors) without any safety issues (2). Despite the promising data presented herein, we have not yet determined whether BM-DC or AS-ODN BM-DC can “reverse,” or inhibit already-established colitis. This step along with validation of the approach in at least two other mouse models of colitis are important milestones before considering autologous tolerogenic DC therapy for IBD. Intriguingly, Badami and colleagues have demonstrated a significant reduction in suppressive Foxp3+ Treg frequency in duodenal biopsies of T1DM patients compared with healthy controls. Furthermore, the patients exhibited an impairment in peripheral blood-derived CD103+ DC to convert CD4+ CD25− T-cells into Foxp3+ Tregs, unlike DC from healthy normal controls (122). These data, along with the accumulating evidence demonstrating a central role for RA-producing DC in regulating Tregs and their frequency, and a significant association between T1DM and IBD, further compel a more incisive investigation into the RA-producing DC/Treg/Breg axis in the gut and the consideration of stably suppressive tolerogenic DC therapy as a cell-based, personalized medicine approach toward attenuating or completely reversing colitis and possibly other IBDs.

Our data show that the measurable outcomes between BM-DC and AS-ODN BM-DC are not different, even though they are significantly different when compared with untreated mice. This is not surprising as BM-DC are well known to be inherently tolerogenic (123–127). The original work using GM-CSF/IL-4 to propagate a DC population from bone marrow progenitors, two decades ago, clearly established that BM-DC, as functionally immature cells in their ability to stimulate significant T-cell proliferation, induced donor-specific hyporesponsiveness to alloantigens in transplantation models (123–127) and also were able to prevent the onset of autoimmune disease, T1D in particular (128–131). Given that the experimental conditions in all these studies used animals in specific pathogen-free facilities, and well-controlled environmental conditions, the reality in natural environments is expected to be much different; whereas the probability of conversion of the BM-DC *in vivo* into immunostimulatory cells under a controlled environment is low, the reality in the wild would predict that the metastable state of BM-DC would be sensitive to stimuli that confer to them a powerful immunostimulatory capacity. For example, a pathogenic enteric infection is expected to convert exogenously administered BM-DC, which accumulate into the gastrointestinal lymph nodes, into proinflammatory DC. Instead, the AS-ODN BM-DC are designed specifically to be co-stimulation impaired, even in conditions that can stimulate a maturation process. The same rationale underlies other approaches to generate tolerogenic DC; to maintain the cells in a state where, even though they may migrate through, or accumulate inside an immunostimulatory environment, the *ex vivo* conditioning maintains at least one major feature that maintains the balance in favor of a tolerogenic state.

## Ethics Statement

All mice were maintained in a specific pathogen-free environment, and experiments were conducted in line with specific protocols approved by the Allegheny Health Network IACUC. The human DC were generated from commercially purchased blood (exempt from IRB requirements).

## Author Contributions

CE, YG, and BP conducted the experimental work. MT critically reviewed the manuscript for important intellectual content. NG was responsible for the design, the interpretation, and the overall compilation of this manuscript.

## Conflict of Interest Statement

NG and MT hold equity in Diavacs Inc., which has licensed the intellectual property (all patents) related to the methods in the preparation of the tolerogenic DC (AS-ODN BM-DC) described herein, as well as the use of the AS-ODN BM-DC and the human counterpart in autoimmune diseases, including colitis and related inflammatory bowel disease. They also serve as members of the Scientific Advisory Body. Diavacs did not review nor did it contribute to any aspect of the research presented in, or the preparation/editing of this manuscript. The reviewer CH and the handling Editor declared their shared affiliation. The reviewer CH declared a past co-authorship with one of the authors NG to the handling Editor.

## Supplementary Material

Figure S1 Verification of AS-ODN BM-dendritic cell (DC) functionality *in vitro*. **(A)** Day 6 culture AS-ODN BM-DC (referred to also as AS-ODN DC in the graph) express significantly less surface CD40, CD80, and CD86 compared with paired BM-DC (cells generated from the same batch of bone marrow progenitors) measured by flow cytometry. The bars show the mean fluorescence intensity (MFI) corresponding to the levels of each of the indicated co-stimulatory proteins. The error bars show the SEM of triplicate measurements in 1 × 10^4^ cells. The differences between the means are statistically significant when analyzed by two-tailed *t*-test. These outcomes are representative of the results obtained when characterizing the DC before administration in mice. **(B)** AS-ODN BM-DC do not stimulate IFNγ production in allogeneic mixed leukocyte culture compared to BM-DC *in vitro*. Two replicate co-cultures are shown (labeled 1 and 2); BM-DC (row of wells on the left side) and AS-ODN BM-DC (row of wells on the right side). These outcomes are representative of the results obtained when characterizing the DC prior to administration in mice.

Figure S2 Regulatory B-cells (Bregs) retrieved from the mesenteric lymph node (MLN) of AS-ODN BM-dendritic cell (DC) recipients exhibit increased levels of IL-10 protein on a per-cell basis. The graph represents the geometric mean fluorescence intensity of the flow cytometry-measured events shown in the histograms in the panels in the middle of Figure 3A. These histograms correspond to IL-10 in permeabilized Bregs obtained from the MLN of PBS-injected mice alone, or mice that were dextran sodium sulfate (DSS)-treated alone, or DSS+ BM-DC or DSS+ AS-ODN BM-DC (2 × 10^6^ cells per mouse) i.p. The analysis for IL-10 events is performed in cells gated into CD11c− CD19+ B220+ populations. The error bars represent the median of four mouse recipients per treatment group. The differences between AS-ODN BM-DC and BM-DC recipients are statistically significant as shown in the graph (repeated-measures, Kruskal–Wallis test).

Figure S3 BM-dendritic cell (DC) and AS-ODN BM-DC accumulate inside the mesenteric lymph node (MLN) of dextran sodium sulfate (DSS)-treated mice following i.p. injection. Flow cytometry analysis to measure the frequency of exogenously administered DC inside the MLN. The panels are representative of an analysis conducted on single cells from freshly collected MLN at 3-h post-injection. The graph represents the frequency (Fluosphere+ CD45+ CD11c+ gated cells as a percentage of total cells) of the DC measurable from the single cells of freshly collected MLN 3-h post-DC injection. The data in the graph are shown as medians in the frequency of cells from the MLN of individual mice (*n* = 3 per treatment group) together with the range. There are no statistically distinguishable differences in accumulation of BM-DC compared with AS-ODN BM-DC; however, the differences in DC accumulation between mice that were administered DC vs. those that were not was statistically relevant (one-way ANOVA).

Figure S4 Human PBMC-derived dendritic cell (DC) generated in the presence of GM-CSF/IL-4 alone (CP-DC) as well as with a mixture of antisense DNA oligonucleotides targeting the primary transcripts of CD40, CD80, and CD86 [tolerogenic human DC (TH-DC)] produce IL-37 *in vitro*. A human-specific IL-37 ELISA (R&D Systems) was used to measure the concentration of the cytokine in the culture supernatants of 1 × 10^5^ CP-DC and TH-DC DC that remained naive or were stimulated with 2 μg/mL LPS overnight (18 h). The graph shows the means of quadruplicate wells of supernatants collected 18 h following DC plating (naive cells) or 18 h following LPS stimulation. The error bars represent the SEM. The differences in IL-37 produced between naive and LPS-stimulated moDC are statistically significant (*p* < 0.02, one-way ANOVA). The differences in IL-37 production between naive TH-DC and LPS-stimulated TH-DC are not statistically significant, even though there is a trend suggestive of more cytokine production by LPS-stimulated iDC. IL-37 production between naive CP-DC and TH-DC are not-significant. IL-37 was not detected in serum-containing, cell-free medium (data not shown).
