# Supplementary figures and images for: Co-Stimulation-Impaired Bone Marrow-Derived Dendritic Cells Prevent Dextran Sodium Sulfate-Induced Colitis in Mice
Source: Front Immunol. 2018 May 3;9:894. doi: 10.3389/fimmu.2018.00894 (PMC5943510; doi:10.3389/fimmu.2018.00894)

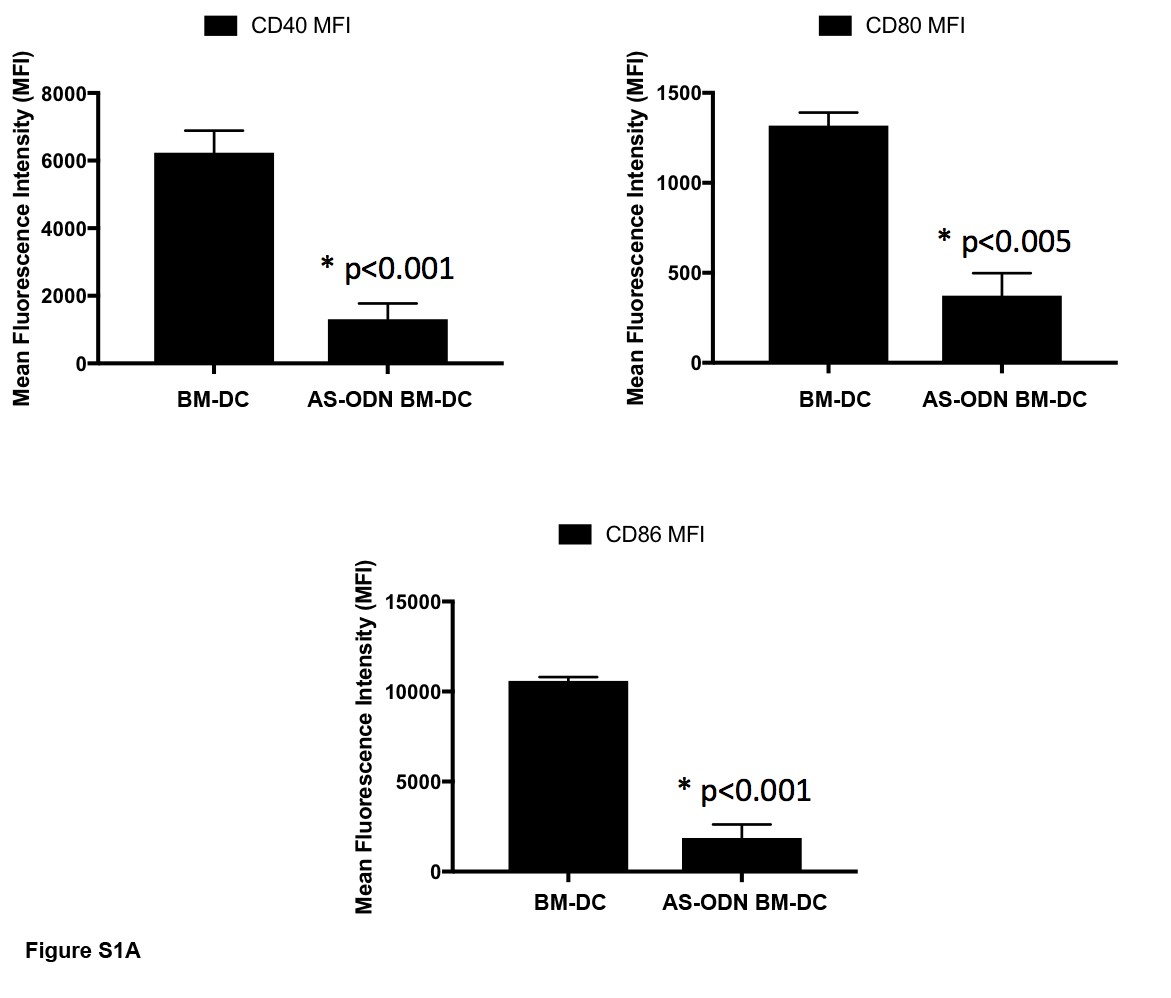

Supplement: Figure S1 — Verification of AS-ODN BM-dendritic cell (DC) functionality in vitro. (A) Day 6 culture AS-ODN BM-DC (referred to also as AS-ODN DC in the graph) express significantly less surface CD40, CD80, and CD86 compared with paired BM-DC (cells generated from the same batch of bone marrow progenitors) measured by flow cytometry. The bars show the mean fluorescence intensity (MFI) corresponding to the levels of each of the indicated co-stimulatory proteins. The error bars show the SEM of triplicate measurements in 1 × 10^4^ cells. The differences between the means are statistically significant when analyzed by two-tailed t-test. These outcomes are representative of the results obtained when characterizing the DC before administration in mice. (B) AS-ODN BM-DC do not stimulate IFNγ production in allogeneic mixed leukocyte culture compared to BM-DC in vitro. Two replicate co-cultures are shown (labeled 1 and 2); BM-DC (row of wells on the left side) and AS-ODN BM-DC (row of wells on the right side). These outcomes are representative of the results obtained when characterizing the DC prior to administration in mice. [file Image_11.TIF]

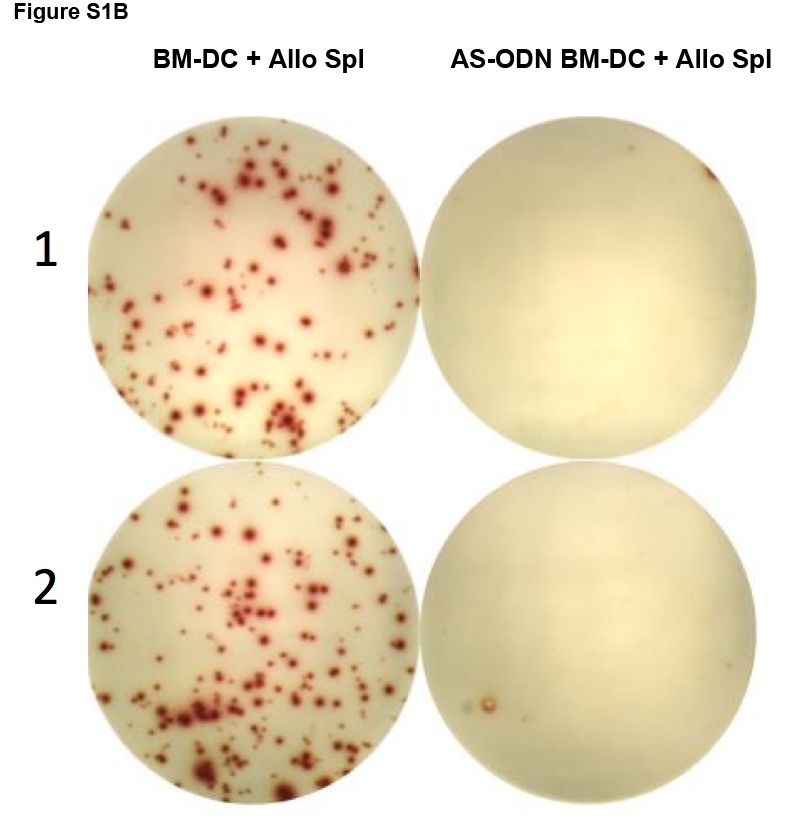

Supplement: Figure S2 — Regulatory B-cells (Bregs) retrieved from the mesenteric lymph node (MLN) of AS-ODN BM-dendritic cell (DC) recipients exhibit increased levels of IL-10 protein on a per-cell basis. The graph represents the geometric mean fluorescence intensity of the flow cytometry-measured events shown in the histograms in the panels in the middle of Figure 3A. These histograms correspond to IL-10 in permeabilized Bregs obtained from the MLN of PBS-injected mice alone, or mice that were dextran sodium sulfate (DSS)-treated alone, or DSS+ BM-DC or DSS+ AS-ODN BM-DC (2 × 10^6^ cells per mouse) i.p. The analysis for IL-10 events is performed in cells gated into CD11c− CD19+ B220+ populations. The error bars represent the median of four mouse recipients per treatment group. The differences between AS-ODN BM-DC and BM-DC recipients are statistically significant as shown in the graph (repeated-measures, Kruskal–Wallis test). [file Image_12.TIF]

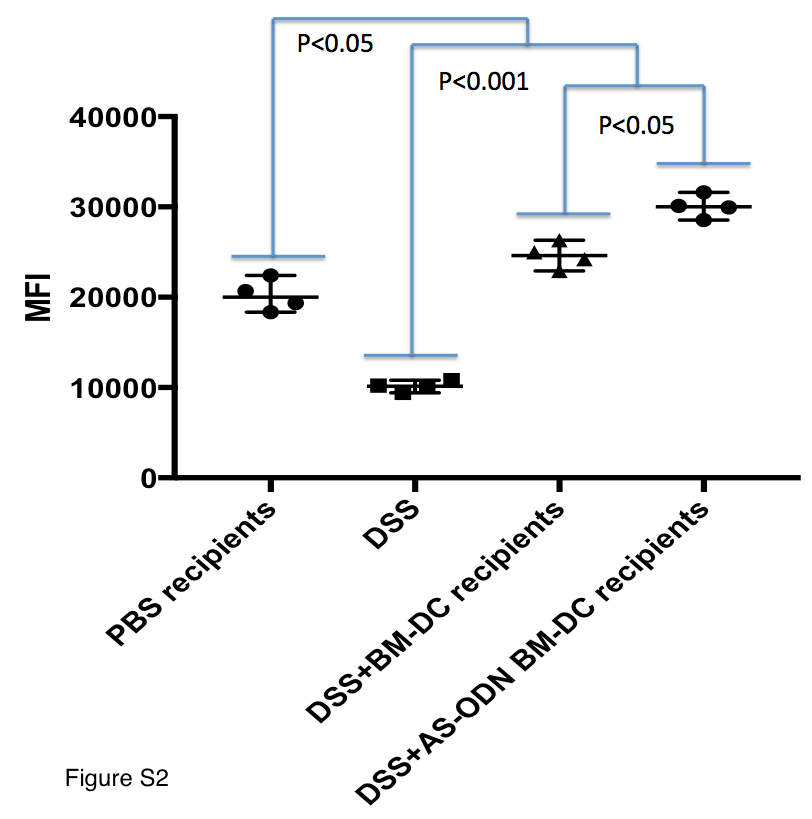

Supplement: Figure S3 — BM-dendritic cell (DC) and AS-ODN BM-DC accumulate inside the mesenteric lymph node (MLN) of dextran sodium sulfate (DSS)-treated mice following i.p. injection. Flow cytometry analysis to measure the frequency of exogenously administered DC inside the MLN. The panels are representative of an analysis conducted on single cells from freshly collected MLN at 3-h post-injection. The graph represents the frequency (Fluosphere+ CD45+ CD11c+ gated cells as a percentage of total cells) of the DC measurable from the single cells of freshly collected MLN 3-h post-DC injection. The data in the graph are shown as medians in the frequency of cells from the MLN of individual mice (n = 3 per treatment group) together with the range. There are no statistically distinguishable differences in accumulation of BM-DC compared with AS-ODN BM-DC; however, the differences in DC accumulation between mice that were administered DC vs. those that were not was statistically relevant (one-way ANOVA). [file Image_13.TIF]

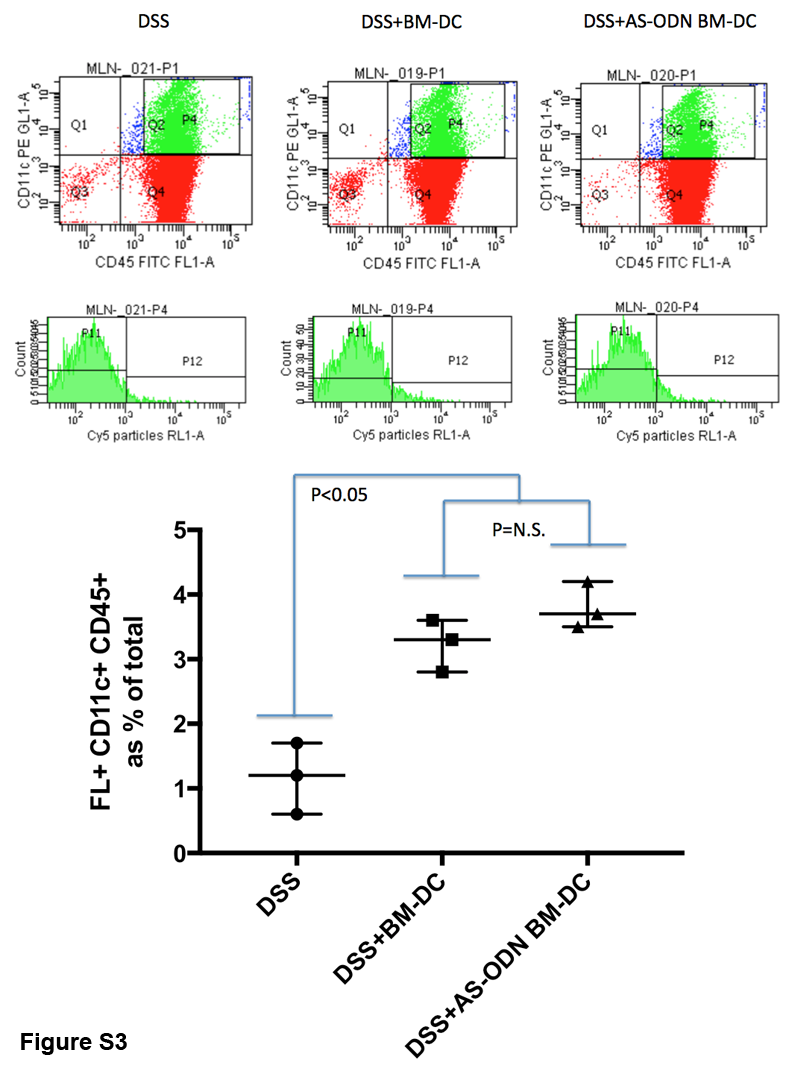

Supplement: Figure S4 — Human PBMC-derived dendritic cell (DC) generated in the presence of GM-CSF/IL-4 alone (CP-DC) as well as with a mixture of antisense DNA oligonucleotides targeting the primary transcripts of CD40, CD80, and CD86 [tolerogenic human DC (TH-DC)] produce IL-37 in vitro. A human-specific IL-37 ELISA (R&D Systems) was used to measure the concentration of the cytokine in the culture supernatants of 1 × 10^5^ CP-DC and TH-DC DC that remained naive or were stimulated with 2 μg/mL LPS overnight (18 h). The graph shows the means of quadruplicate wells of supernatants collected 18 h following DC plating (naive cells) or 18 h following LPS stimulation. The error bars represent the SEM. The differences in IL-37 produced between naive and LPS-stimulated moDC are statistically significant (p < 0.02, one-way ANOVA). The differences in IL-37 production between naive TH-DC and LPS-stimulated TH-DC are not statistically significant, even though there is a trend suggestive of more cytokine production by LPS-stimulated iDC. IL-37 production between naive CP-DC and TH-DC are not-significant. IL-37 was not detected in serum-containing, cell-free medium (data not shown). [file Image_14.TIF]

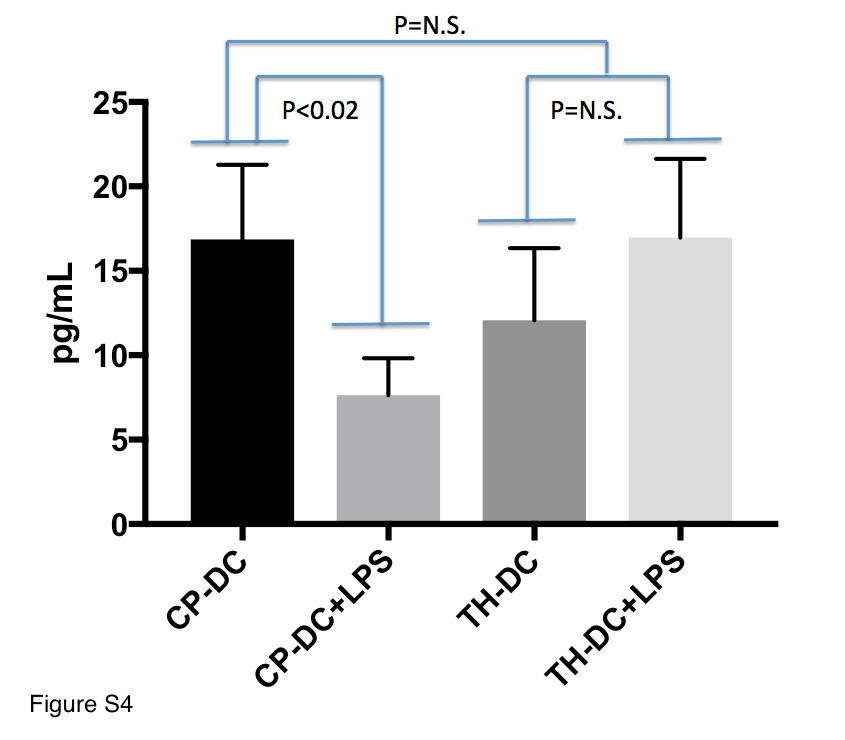

Supplement: Supplementary file 5 [file Image_15.TIF]
